# Nasal Discharge in Children

**Published:** 1907-05-18

**Authors:** 


### Nose and Throat.

### NASAL DISCHARGE IN CHILDREN.

One of the commonest and most important complaints of childhood is the presence of a nasal discharge. This should never be neglected, as it always means that, if unrelieved, there will be obstruction to nasal respiration, and connotes the possibility that the inflammatory process may extend backwards into the naso-pharynx and thence along the Eustachian tubes to the tympanum. A large proportion of the cases of middle-ear disease in children are the direct result of a neglected chronic rhinitis. A nasal discharge may be due to a variety of causes, and for its successful treatment the cause must first be sought. To investigate such a case we must ascertain whether the trouble is acute or chronic, and whether the discharge is continuous or intermittent. Next it is necessary to notice the character of the discharge, whether mucoid, muco-purulent, or purulent, and inquiries should be made as to whether it is ever blood-stained. The presence of any soreness or excoriation of the nostril or lip should also be noted.

Acute catarrhal rhinitis gives rise to a discharge mucoid or muco-purulent in character, and though this may be only that of an ordinary cold, yet it will be remembered that it may signify the onset of measles or influenza. More important is acute purulent rhinitis, which usually occurs in infants shortly after birth, and is caused by infection of the nasal mucous membrane by gonorrhœal or leucorrhœal discharge. This is a serious trouble, for the discharge of pus is associated with swelling of the mucous membrane, leading to so much nasal obstruction that the child may be unable to take the breast and so require spoon feeding. This disease must be distinguished from the early nasal discharge which occurs in congenital syphilis. The latter occurs later, is less acute, and the discharge is muco-purulent in character. Gonorrhœal rhinitis should be treated by gently irrigating the nose with warm boracic lotion, and afterwards painting the nasal mucous membrane with a 1 per cent. solution of protargol. Purulent rhinitis may also occur in measles and in certain other infectious diseases. In the more chronic forms of rhinitis the discharge may be unilateral or bilateral. When unilateral, and especially when the discharge is muco-purulent, occasionally blood-stained, and excoriates the lip, a foreign body should be suspected. This may often be detected by anterior rhinoscopy, or if the discharge prevents a satisfactory view of the nasal fossa, may be felt by a probe. A foreign body is best removed by passing a director along the floor of the nose well behind it. That part of the director outside the nose is then depressed, and the director is drawn out in this position. The foreign body is thus hooked forwards and appears at the nostril. The removal of a foreign body should never be attempted by syringing, as this is very likely to force septic material along the Eustachian tube into the middle ear.

Nasal diphtheria is an occasional cause of a unilateral nasal discharge. This disease may occur in several forms. Thus it may be secondary to ordinary faucial diphtheria by direct extension. It is then a very serious complication, though the diagnosis will present no difficulties. Diphtheria may, however, be primary in the nose, and when this is so it is usually chronic and causes but little constitutional disturbance. The discharge is mucoid, or muco-purulent, is frequently blood-stained, and causes soreness and excoriation of the upper lip. A definite membrane may be present, though in a large number of cases it is absent. Both nasal fossæ may be affected, or the discharge may be much more profuse from one nostril. Not infrequently it is found that the child has lately had scarlet fever, and that the discharge appeared during convalescence. When a child previously healthy has for some weeks suffered from a nasal discharge with the characters described above, for which no obvious cause can be found, a cultivation should always be taken. These cases show the typical Klebs Loeffler bacillus, though its virulence is comparatively slight, as is shown by the trivial constitutional disturbance and the fact that in the post-scarlatinal variety it seldom gives rise to faucial diphtheria. The following is a good example of unilateral nasal diphtheria. A few weeks ago a child, aged 4, was brought to the throat out-patient department at Guy's Hospital suffering from a muco-purulent nasal discharge of some weeks' duration. A foreign body was suspected, but on examination with a probe a large piece of typical membrane was brought away. A cultivation showed typical diphtheria bacilli, and the child was removed to a fever hospital. In treating these cases gentle irrigation of the nasal fossæ with lot. pot. permang. (gr. ij. ad ʒj.) or dilute hydrarg. perchlorid. lotion (1-5,000) should be used as well as the injection of antitoxin.

Suppuration in the maxillary antrum, a common cause of a unilateral purulent discharge in adults, is rare in children, though it has been recorded even in infants. The discharge in these cases consists of foul yellow pus. The methods of diagnosis and treatment are the same as in adults. A discharge of pus from one or both nostrils also occurs with an abscess of the nasal septum, usually the result of injury.

An extremely important cause of chronic rhinitis is the presence of adenoids in the naso-pharynx. The discharge may be continuous or it may vary from time to time. The history given is frequently that the child is extremely liable to colds, which it has a great difficulty in shaking off. The discharge is usually clear and mucoid, though it may be muco-purulent and blood-stained; it is commonly bilateral, and sometimes one nasal fossa is affected far more than the other. Before a unilateral muco-purulent blood-stained discharge is ascribed to adenoids, the presence of a foreign body and of nasal diphtheria must both be excluded. Last autumn a child was sent to the throat department at Guy's for removal of adenoids and enlarged tonsils. These were certainly present, but it also had a typical unilateral blood-stained discharge. Examination with a director revealed the presence of a roll of paper in the nasal fossa, which, from its appearance, must have been there many weeks.

A chronic rhinitis associated with the presence of adenoids, even of a small mass, is a strong indication for removal of these growths; for the inflammatory process is very likely to extend back into the naso-pharynx and cause a suppurative otitis media. Removal of the adenoids, followed by systematic breathing exercises, usually soon cures the rhinitis. Chronic rhinitis may also occur as a sequela of certain infectious diseases, such as measles, scarlet fever, and influenza, especially when the child is not well looked after during convalescence. As the result of a long continued rhinitis the mucous membrane becomes thickened and hyptertrophied, especially over the inferior turbinated bones; much thick mucus is secreted, and considerable nasal obstruction results. This condition is known as hypertrophic rhinitis, and though in children removal of the original cause of the trouble, combined with tonics and local treatment, will often effect a cure, yet in older children it may be necessary also to treat directly the enlarged turbinates, either by the galvano cautery or by partial removal.

Atrophic rhinitis usually commences about the time of puberty, but may occur much earlier. It is characterised by atrophy of the nasal mucous membrane associated with the presence of greenish inspissated crusts, and of an extremely foul discharge from the nostrils. Its relation to the other forms of rhinitis is doubtful, though it certainly sometimes follows a simple chronic rhinitis. Congenital syphilis is an extremely important cause of chronic nasal discharge. It may occur in two forms: (1) "Snuffles," which occurs in infants a few weeks old, and is due to a catarrhal inflammation of the mucous membrane without ulceration, and without necrosis of cartilage or bone. The onset is gradual, the mucous membrane is much swollen, and the mucoid discharge is irritating, and causes fissures and ulceration of the nostril. Owing to the obstruction the child may not be able to suck, rendering spoon feeding necessary until the disease has been controlled by the administration of small doses of hydrarg. c. cret. (2) Gummatous affections of the nasal fossæ, which occur usually between the ages of five and fifteen. The gummata break down and lead to extensive ulceration and necrosis, especially of the septum and of the turbinated bones. There is a profuse discharge of very foul pus, and owing to the necrosis considerable deformity may result. There is usually no difficulty in diagnosis, though this condition may occasionally be confused with atrophic rhinitis, but it must be remembered that no ulceration occurs in the latter disease. The treatment consists in the internal administration of mercury and potassium iodide; locally, the nose should be douched with alkaline and antiseptic lotions, and whenever a sequestrum becomes loose it should be gently removed.

